# Branebrutinib (BMS-986195), a Bruton’s Tyrosine Kinase Inhibitor, Resensitizes P-Glycoprotein-Overexpressing Multidrug-Resistant Cancer Cells to Chemotherapeutic Agents

**DOI:** 10.3389/fcell.2021.699571

**Published:** 2021-07-19

**Authors:** Chung-Pu Wu, Megumi Murakami, Yu-Shan Wu, Ya-Chen Chi, Sung-Han Hsiao, Yang-Hui Huang, Tai-Ho Hung, Suresh V. Ambudkar

**Affiliations:** ^1^Graduate Institute of Biomedical Sciences, College of Medicine, Chang Gung University, Taoyuan City, Taiwan; ^2^Department of Physiology and Pharmacology, College of Medicine, Chang Gung University, Taoyuan City, Taiwan; ^3^Department of Obstetrics and Gynecology, Taipei Chang Gung Memorial Hospital, Taipei, Taiwan; ^4^Laboratory of Cell Biology, Center for Cancer Research, National Cancer Institute, NIH, Bethesda, MD, United States; ^5^Department of Chemistry, Tunghai University, Taichung, Taiwan; ^6^Department of Medicine, College of Medicine, Chang Gung University, Taoyuan City, Taiwan

**Keywords:** multidrug resistance, chemotherapy, P-glycoprotein, ABCB1, branebrutinib, BMS-986195

## Abstract

The overexpression of P-glycoprotein (P-gp/ABCB1), an ATP-binding cassette (ABC) drug transporter, often contributes to the development of multidrug resistance (MDR) in cancer cells. P-gp mediates the ATP hydrolysis-dependent efflux of a wide range of chemotherapeutic agents out of cancer cells, thereby reducing the intracellular drug accumulation and decreasing the chemosensitivity of these multidrug-resistant cancer cells. Studies with tyrosine kinase inhibitors (TKIs) in P-gp-overexpressing cells have shown that certain TKIs could reverse MDR mediated by P-gp, while some TKIs are transported by P-gp. In the present work, we explored the prospect of repositioning branebrutinib (BMS-986195), a highly selective inhibitor of Bruton’s tyrosine kinase (BTK), to resensitize P-gp-overexpressing multidrug-resistant cancer cells to chemotherapeutic agents. Our results demonstrated that branebrutinib is capable of reversing P-gp-mediated MDR at sub-toxic concentrations, most likely by directly inhibiting the drug transport function of P-gp. Our findings were supported by the result of branebrutinib stimulating the ATPase activity of P-gp in a concentration-dependent manner and the *in silico* study of branebrutinib binding to the substrate-binding pocket of P-gp. In addition, we found that branebrutinib is equally cytotoxic to drug-sensitive parental cell lines and the respective P-gp-overexpressing multidrug-resistant variants, suggesting that it is unlikely that the overexpression of P-gp in cancer cells plays a significant role in reduced susceptibility or resistance to branebrutinib. In summary, we discovered an additional pharmacological action of branebrutinib against the activity of P-gp, which should be investigated further in future drug combination studies.

## Introduction

P-glycoprotein (P-gp or ABCB1) is the most well-characterized member of the human ATP-binding cassette (ABC) transporter family that has been linked to the development of multidrug resistance (MDR) in cancer (Gottesman and Ambudkar, 2001; Robey et al., 2018). P-gp uses energy derived from ATP hydrolysis to actively efflux structurally unrelated chemotherapeutic drugs out of cancer cells and reduces the intracellular accumulation of these drugs. Some of the most well-known P-gp substrate drugs include *Vinca alkaloids*, paclitaxel, colchicine, and anthracyclines (Ambudkar et al., 1999; Gottesman et al., 2002). Consequently, the overexpression of P-gp in cancer cells frequently contributes to reduced chemosensitivity, treatment failure, and recurrence in cancer patients (Szakacs et al., 2006; Wu et al., 2011; Robey et al., 2018). In particular, the link between the high expression of P-gp and poor clinical outcome has been reported in patients with metastatic breast cancer (MBC) (Kovalev et al., 2013) and blood cancers (Ross, 2000; Robey et al., 2018) such as chronic lymphocytic leukemia (CLL) (Matthews et al., 2006), chronic myeloid leukemia (CML) (Maia et al., 2018), and multiple myeloma (MM) (Schwarzenbach, 2002; Tsubaki et al., 2012). Therefore, the discovery and development of P-gp modulators for clinical use is of great significance.

For years, the advancement of P-gp inhibitors has not been successful, which is frequently due to unforeseen toxicities and adverse drug-drug interactions (Dong et al., 2020). For example, tariquidar (XR9576) was developed as a selective and potent inhibitor of P-gp (Mistry et al., 2001), capable of increasing P-gp substrate drug accumulation in drug-resistant tumors (Agrawal et al., 2003). Unfortunately, unexpected toxicity caused two phase III clinical trials of vinorelbine combined with tariquidar (ClinicalTrials.gov Identifier: NCT00042315) and paclitaxel/carboplatin combined with tariquidar (NCT00042302) as first-line therapy in non-small cell lung cancer (NSCLC) to terminate prematurely. To date, the US Food and Drug Administration (FDA) has not approve any agent for the treatment of multidrug-resistant cancers.

As an alternative to developing novel inhibitors, the drug repositioning approach has been exploited by various research groups to utilize tyrosine kinase inhibitors (TKIs) against P-gp-mediated MDR (Beretta et al., 2017). As the result, some well-known TKIs such as osimertinib (Hsiao et al., 2016) and midostaurin (Hsiao et al., 2019), were identified as drug candidates for resensitizing P-gp-overexpressing cancer cells to chemotherapeutic agents (Wu and Fu, 2018). Branebrutinib (BMS-986195) is an orally available, highly selective inhibitor of Bruton’s tyrosine kinase (BTK) (Watterson et al., 2019; Zheng et al., 2019). The safety, tolerability and pharmacokinetics of branebrutinib in healthy participants (Catlett et al., 2020), both as a single drug or in combination with other therapeutic agents, have been studied in several clinical trials (NCT03245515, NCT02705989, NCT03262740, NCT03131973). Currently, branebrutinib is being evaluated as monotherapy in clinical trials in patients with moderate to severe psoriasis (NCT02931838), or active systemic Lupus Erythematosus or Primary Sjögren’s Syndrome (NCT04186871).

In the present study, we found that branebrutinib could inhibit P-gp-mediated drug transport and consequently resensitize P-gp-overexpressing multidrug-resistant cancer cells to apoptosis and cytotoxicity induced by P-gp substrate drugs. Moreover, we observed that P-gp-overexpressing cell lines do not confer significant resistance to branebrutinib as compared to their respective parental cell lines. In summary, our study revealed an additional pharmacological action of branebrutinib, which could potentially be utilized in combination therapies against multidrug-resistant cancers and warrant further studies.

## Materials and Methods

### Chemicals

All culture media and supplements were obtained from Gibco, Invitrogen (Carlsbad, CA, United States). Tools Cell Counting (CCK-8) kit was purchased from Biotools Co., Ltd. (Taipei, Taiwan). Annexin V FITC-Apoptosis Detection Kit was obtained from BD Pharmingen (San Diego, CA, United States). Primary and secondary antibodies were purchased from Abcam (Cambridge, MA, United States). Branebrutinib was purchased from Selleckchem (Houston, TX, United States). Tariquidar (XR9576) and all other chemicals were purchased from Sigma (St. Louis, MO, United States) unless stated otherwise.

### Cell Lines

Human embryonic kidney 293 cells (HEK293) and P-gp-transfected HEK293 cells (MDR19-HEK293); the mouse NIH3T3 and P-gp -transfected NIH3T3-G185 fibroblast cells; the human epidermal cancer cell line KB-3-1 and its P-gp-overexpressing variant KB-V-1 were maintained in Dulbecco’s Modified Eagle’s Medium (DMEM). The human ovarian cancer cell line OVCAR-8 and its P-gp-overexpressing variant NCI-ADR-RES; the human myelogenous leukemia K562 and the P-gp-expressing K562/i-S9 cell lines were maintained in Roswell Park Memorial Institute (RPMI-1640) medium (Mechetner et al., 1997). KB-V-1 cells were maintained in the presence of 1 μg/mL of vinblastine (Shen et al., 1986), NIH3T3-G185 cells were maintained in the presence of 60 ng/mL colchicine (Currier et al., 1992), whereas the HEK293 transfectants were maintained in the presence of 2 mg/mL of G418 (Wu et al., 2007). All cells were cultured at 37°C in 5% CO_2_ humidified air and grown in media supplemented with 10% FCS, L-glutamine and 100 units/mL of penicillin and streptomycin. Cells were maintained in a drug-free medium for 7 days before assay.

### Cytotoxicity Assay

Cytotoxicity was measured with MTT and Cell Counting Kit-8 (CCK-8) assays. Cultured cells were seeded in 96-well flat-bottom plates and allowed to attach for 24 h before incubated in increasing concentrations of a single drug or drug combination for an additional 72 h. Cytotoxicity (IC_50_ value) of each drug regimen was calculated using fitted concentration-response curve from at least three independent experiments. The extent of drug resistance was presented as a resistance-factor (RF) value, whereas the chemosensitizing effect was presented as a fold-reversal (FR) value as previously described (Wu et al., 2007; Dai et al., 2008).

### Apoptosis Assay

The concurrent annexin V–FITC and propidium iodide (PI) staining method was used to determine the effect of branebrutinib on colchicine-induced apoptosis in cancer cells as previously described (Anderson et al., 2003). Briefly, cells were treated with either DMSO, branebrutinib, a known apoptosis inducer colchicine, or the combination of colchicine and branebrutinib for 48 h. Cells were subsequently stained with 1.25 μg/mL of annexin V–FITC and 0.1 mg/mL of PI for 15 min at room temperature, and analyzed using the FACSCalibur flow cytometer equipped with CellQuest software (Becton-Dickinson Biosciences, San Jose, CA, United States) as previously described (Hsiao et al., 2016).

### Flow Cytometry

The intracellular accumulation of the fluorescent P-gp substrate drug calcein (Hollo et al., 1994) was determined in the presence of DMSO (control), branebrutinib, or verapamil using the FACSCalibur flow cytometer, and analyzed using the CellQuest or FlowJo software (Tree Star, Inc., Ashland, OR, United States) software according to the method described by Gribar et al. (2000) and as previously described (Robey et al., 2004; Wu et al., 2013).

### Immunoblotting

Cells were treated with either DMSO (control) or branebrutinib (1–10 μM) for 72 h before being harvested and subjected to SDS-polyacrylamide electrophoresis and immunoblotting as described previously (Wu et al., 2007). Primary antibodies C219 (1:3,000 dilution), anti-alpha tubulin (1:100,000 dilution) (Abcam, Cambridge, MA, United States) and the secondary horseradish peroxidase-conjugated goat anti-mouse IgG (1:100,000 dilution) were used to detect P-gp and the positive loading control tubulin, respectively. The enhanced chemiluminescence (ECL) kit was used for signal detection (Merck Millipore, Billerica, MA, United States).

### ATPase Assay

ATPase activity of P-gp in total membranes prepared from High-Five insect cells (Invitrogen, Carlsbad, CA, United States) infected with recombinant baculovirus carrying the *MDR*1 gene was measured by endpoint inorganic phosphate (P_*i*_) assay [33] and recorded as vanadate (Vi)-sensitive ATPase activity as previously described (Ambudkar, 1998; Wu et al., 2019). GraphPad Prism software (GraphPad Software, La Jolla, CA, United States) was used to calculate the EC_50_ values based on fitted concentration-response curves obtained from three independent experiments as previously described (Wu et al., 2019).

### *In silico* Analysis of Docking of Branebrutinib in the Drug-Binding Pocket of P-gp

The cryo-EM structure of P-gp was obtained from the Protein Data Bank (PDB:6QEX) and the protein was prepared by addition of hydrogen atoms and partial charges based on CHARMM force field at pH of 7.4 using Accelrys Discovery Studio 4.0 (Alam et al., 2019). Branebrutinib structure was optimized and docking was performed by the CDOCKER module of the same software. The conformation with the lowest CDOCKER Interaction Energy was selected and the respective interaction energy was calculated.

### Quantification and Statistical Analysis

Data are presented as mean ± standard deviation (SD) from at least three independent experiments unless stated otherwise. Statistical analysis was performed using KaleidaGraph software (Synergy Software, Reading, PA, United States). Two-tailed Student’s *t*-test was performed to analyze the difference between mean values of experimental and control or improvement in fit and labeled with asterisks as “statistically significant” if the probability, *p*, was less than 0.05.

## Results

### Branebrutinib Resensitizes P-gp-Overexpressing Multidrug-Resistant Cells to Cytotoxic Therapeutic Agents

**FIGURE 1:**
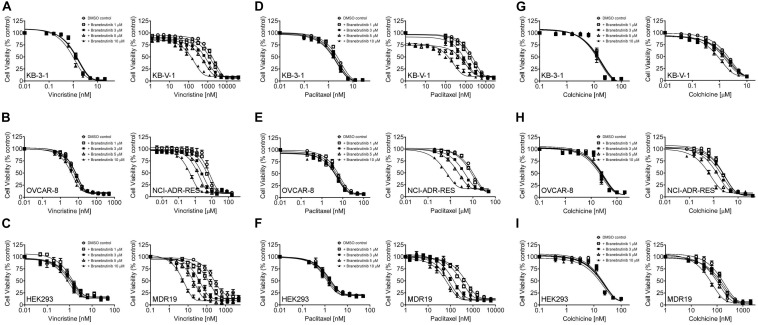
Branebrutinib reverses multidrug resistance (MDR) in P-glycoprotein (P-gp) -overexpressing cells. The effect of branebrutinib on P-gp-mediated resistance to vincristine **(A–C)**, paclitaxel **(D–F)** and colchicine **(G–I)** was tested in drug-sensitive parental human KB-3-1 epidermal cancer cell line **(A,D,G-left panel)** and its P-gp-overexpressing multidrug-resistant KB-V-1 subline **(A,D,G-right panel)**; drug-sensitive parental human OVCAR-8 ovarian cancer cell line **(B,E,H-left panel)** and its P-gp-overexpressing multidrug-resistant NCI-ADR-RES subline **(B,E,H-right panel)**; as well as parental HEK293 cells **(C,F,I-left panel)** and MDR19-HEK293 cells, which are HEK293 cells transfected with human P-gp **(C,F,I-right panel)**. Cells were treated with increasing concentration of vincristine, paclitaxel or colchicine in the presence of DMSO (open circles) or branebrutinib at 1 μM (open squares), 3 μM (filled squares), 5 μM (open triangles), or at 10 μM for 72 h before analysis as described in Materials and methods. Points, mean values from at least three independent experiments; bars; S.E.M.

We investigated the potential chemosensitizing effect of branebrutinib on P-gp-mediated resistance to known P-gp drug substrates (Kartner et al., 1983) such as vincristine, paclitaxel and colchicine in P-gp-overexpressing multidrug-resistant human epidermal KB-V-1 cancer cells, human ovarian NCI-ADR-RES cancer cells, and P-gp-transfected MDR19-HEK293 cells. We discovered that without significantly affecting the respective drug-sensitive parental cells (Figure 1, left panels), branebrutinib significantly reversed P-gp-mediated resistance to vincristine in KB-V-1 (Figure 1A, right panel), NCI-ADR-RES (Figure 1B, right panel), and MDR19-HEK293 (Figure 1C, right panel) cells in a concentration-dependent manner. Moreover, we found that P-gp-mediated resistance to paclitaxel (Figures 1D–F) and colchicine (Figures 1G–I) in these P-gp-overexpressing multidrug-resistant cells was reversed by branebrutinib in the same manner. The respective IC_50_ values and the extent of chemosensitization by branebrutinib in these cell lines, represented by the fold-reversal (FR) values (Dai et al., 2008), are summarized in Tables 1, 2. The FR value was calculated by dividing the IC_50_ value of a P-gp drug substrate by the IC_50_ value of the same drug substrate in the presence of branebrutinib or the P-gp reference inhibitor verapamil (Dai et al., 2008). Our data show that branebrutinib restores the chemosensitivity of P-gp-overexpressing cells to chemotherapeutic drugs at sub-toxic concentrations.

**TABLE 1:** Chemosensitizing effect of branebrutinib on drug-selected P-glycoprotein (P-gp)-overexpressing human cancer cell lines.

		Mean IC_50_^†^ ± SD and (FR^‡^)	
Treatment	Concentration (μM)	OVCAR-8 (parental) [nM]	NCI-ADR-RES (resistant) [μM]
Vincristine	–	6.48 ± 0.92 (1.0)	6.50 ± 1.19 (1.0)
+ branebrutinib	1	6.12 ± 0.85 (1.1)	5.03 ± 1.01 (1.3)
+ branebrutinib	3	5.21 ± 0.57 (1.2)	2.51 ± 0.39** (2.6)
+ branebrutinib	5	5.23 ± 0.89 (1.2)	1.40 ± 0.24** (4.6)
+ branebrutinib	10	3.92 ± 0.63* (1.7)	0.47 ± 0.07*** (13.8)
+ verapamil	5	1.84 ± 0.30** (3.5)	0.22 ± 0.05*** (29.5)

		**[nM]**	**[μM]**

Paclitaxel	–	4.50 ± 0.81 (1.0)	8.93 ± 1.62 (1.0)
+ branebrutinib	1	3.85 ± 0.53 (1.2)	7.56 ± 1.31 (1.2)
+ branebrutinib	3	4.70 ± 0.68 (1.0)	4.00 ± 0.37** (2.2)
+ branebrutinib	5	3.88 ± 0.53 (1.2)	2.21 ± 0.28** (4.0)
+ branebrutinib	10	4.36 ± 0.79 (1.0)	0.61 ± 0.19*** (14.6)
+ verapamil	5	3.97 ± 0.71 (1.1)	0.57 ± 0.08*** (15.7)

		**[nM]**	**[μM]**

Colchicine	–	18.96 ± 7.09 (1.0)	2.01 ± 0.49 (1.0)
+ branebrutinib	1	18.78 ± 7.34 (1.0)	1.93 ± 0.45 (1.0)
+ branebrutinib	3	18.51 ± 6.85 (1.0)	1.64 ± 0.36 (1.2)
+ branebrutinib	5	17.11 ± 6.61 (1.1)	1.15 ± 0.24 (1.7)
+ branebrutinib	10	15.37 ± 5.51 (1.2)	0.59 ± 0.12** (3.4)
+ verapamil	5	14.13 ± 5.64 (1.3)	0.46 ± 0.12** (4.4)

**Treatment**	**Concentration (μM)**	**KB-3-1 (parental) [nM]**	**KB-V-1 (resistant) [μM]**

Vincristine	–	1.12 ± 0.34 (1.0)	1.72 ± 0.22 (1.0)
+ branebrutinib	1	1.09 ± 0.35 (1.0)	1.33 ± 0.16 (1.3)
+ branebrutinib	3	1.03 ± 0.32 (1.1)	0.80 ± 0.09** (2.2)
+ branebrutinib	5	1.05 ± 0.32 (1.1)	0.29 ± 0.03*** (6.0)
+ branebrutinib	10	0.77 ± 0.22 (1.5)	0.0913 ± 0.001*** (19)
+ verapamil	5	0.24 ± 0.08* (4.7)	0.0481 ± 0.001*** (36)

		**[nM]**	**[μM]**

Paclitaxel	–	1.89 ± 0.49 (1.0)	1.95 ± 0.21 (1.0)
+ branebrutinib	1	1.56 ± 0.38 (1.2)	1.43 ± 0.09* (1.4)
+ branebrutinib	3	1.26 ± 0.28 (1.5)	0.76 ± 0.07*** (2.6)
+ branebrutinib	5	1.52 ± 0.37 (1.2)	0.78 ± 0.17** (2.5)
+ branebrutinib	10	1.34 ± 0.32 (1.4)	0.20 ± 0.04*** (9.8)
+ verapamil	5	1.51 ± 0.40 (1.3)	0.0676 ± 0.002*** (29)
		**[nM]**	**[μM]**
Colchicine	–	10.92 ± 4.08 (1.0)	1.80 ± 0.18 (1.0)
+ branebrutinib	1	12.14 ± 4.69 (0.9)	1.68 ± 0.18 (1.1)
+ branebrutinib	3	11.70 ± 4.60 (0.9)	1.53 ± 0.15 (1.2)
+ branebrutinib	5	11.48 ± 4.63 (1.0)	1.31 ± 0.11* (1.4)
+ branebrutinib	10	10.19 ± 4.07 (1.1)	0.82 ± 0.06*** (2.2)
+ verapamil	5	11.75 ± 4.69 (0.9)	0.67 ± 0.09*** (2.7)

*FR, fold-reversal.*

*^†^IC_50_ values were calculated from at least three independent experiments as described in Materials and methods.*

^‡^ FR value was calculated by dividing the IC_50_ value of a P-gp drug substrate by the IC_50_ value of the same drug substrate in the presence of branebrutinib or the P-gp reference inhibitor verapamil.

*p < 0.05; **p < 0.01; ***p < 0.001.

**TABLE 2:** Chemosensitizing effect of branebrutinib on HEK293 cells transfected with human P-glycoprotein (P-gp).

		Mean IC_50_^†^ ± SD and (FR^‡^)	
Treatment	Concentration (μM)	pcDNA3.1-HEK293 (parental) [nM]	MDR19-HEK293 (resistant) [nM]
Vincristine	–	1.46 ± 0.37 (1.0)	305.07 ± 60.61 (1.0)
+ branebrutinib	1	1.39 ± 0.24 (1.1)	125.26 ± 20.31** (2.4)
+ branebrutinib	3	1.26 ± 0.18 (1.2)	39.70 ± 6.68** (7.7)
+ branebrutinib	5	1.10 ± 0.21 (1.3)	17.73 ± 1.96** (17.2)
+ branebrutinib	10	0.89 ± 0.18 (1.6)	4.94 ± 0.81** (61.8)
+ verapamil	5	0.44 ± 0.10** (3.3)	2.70 ± 0.25*** (113.0)

		**[nM]**	**[nM]**

Paclitaxel	–	2.14 ± 0.56 (1.0)	520.29 ± 49.34 (1.0)
+ branebrutinib	1	2.23 ± 0.48 (1.0)	326.10 ± 33.09** (1.6)
+ branebrutinib	3	2.24 ± 0.47 (0.8)	143.26 ± 20.09*** (3.6)
+ branebrutinib	5	1.75 ± 0.37 (1.2)	86.34 ± 13.74*** (6.0)
+ branebrutinib	10	1.50 ± 0.36 (1.4)	66.06 ± 10.81*** (7.9)
+ verapamil	5	2.22 ± 0.39 (1.0)	32.15 ± 7.43*** (16.2)

		**[nM]**	**[nM]**

Colchicine	–	18.36 ± 5.73 (1.0)	135.76 ± 32.28 (1.0)
+ branebrutinib	1	17.84 ± 5.01 (1.0)	116.26 ± 24.33 (1.2)
+ branebrutinib	3	18.01 ± 5.01 (1.0)	102.40 ± 18.01 (1.3)
+ branebrutinib	5	17.15 ± 4.42 (1.1)	78.81 ± 14.59* (1.7)
+ branebrutinib	10	15.73 ± 3.92 (1.2)	43.65 ± 9.23** (3.1)
+ verapamil	5	17.48 ± 5.16 (1.1)	57.08 ± 13.83* (2.4)

*FR, fold-reversal.*

*^†^IC_50_ values were calculated from at least three independent experiments as described in Materials and Methods.*

^‡^FR value was calculated by dividing the IC_50_ value of a P-gp drug substrate by the IC_50_ value of the same drug substrate in the presence of branebrutinib or the P-gp reference inhibitor verapamil.

*p < 0.05; **p < 0.01; ***p < 0.001.

### Branebrutinib Antagonizes the Drug Efflux Function of P-gp

**FIGURE 2:**
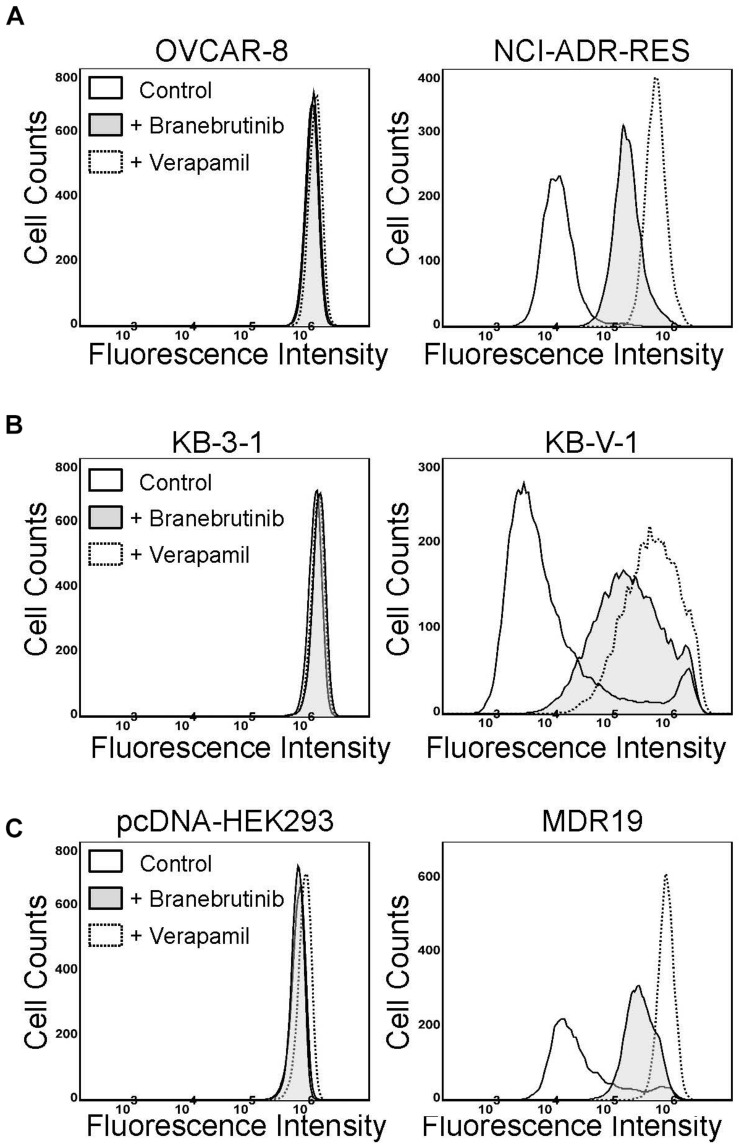
Branebrutinib inhibits P-glycoprotein (P-gp)-mediated drug efflux. The effect of branebrutinib on the intracellular accumulation of calcein, a fluorescent product of a known P-gp substrate calcein-AM, was determined in **(A)** OVCAR-8 (left panel) and NCI-ADR-RES cancer cells (right panel); **(B)** KB-3-1 (left panel) and KB-V-1 (right panel); and **(C)** HEK293 cells (left panel) and P-gp-transfected MDR19-HEK293 cells (right panel). Cells were treated with DMSO (control, solid lines), 20 μM of branebrutinib (filled solid lines) or 20 μM of a reference inhibitor verapamil (dotted lines) as a positive control. The fluorescence signal was analyzed by flow cytometry as described previously (Wu et al., 2007). Representative histograms of at least three independent experiments are shown.

Previous studies have demonstrated that a common way for a modulator to resensitize P-gp-overexpressing cells to P-gp drug substrates is by directly inhibiting the drug efflux function of P-gp (Hsiao et al., 2016, 2018, 2019; Wu et al., 2019, 2020b). To this end, we examined the effect of branebrutinib on the drug transport function of P-gp by performing a short-term fluorescent drug efflux assay in NCI-ADR-RES (Figure 2A) and KB-V-1 (Figure 2B) cancer cells, as well as in MDR9-HEK293 (Figure 2C) cells. Cells were incubated with a P-gp substrate calcein-AM (Hollo et al., 1994) in the presence of DMSO (solid line), or 20 μM of branebrutinib (filled solid line) or 20 μM of verapamil (dotted line), and the intracellular accumulation of calcein, a fluorescent product of calcein-AM, was monitored for 10 min as described in Materials and methods. We discovered that without affecting the accumulation of calcein in drug-sensitive parental cells (Figure 2, left panels), branebrutinib significantly increased the intracellular accumulation of fluorescent calcein in P-gp-overexpressing NCI-ADR-RES, KB-V-1, and MDR9-HEK293 cells (Figure 2, right panels).

**FIGURE 3:**
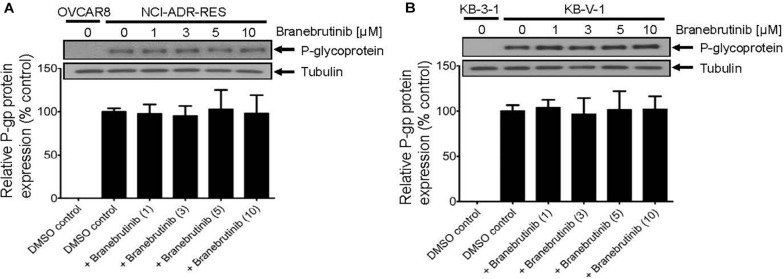
Branebrutinib has no significant effect on the P-glycoprotein (P-gp) protein levels after 72 h exposure. The P-gp-overexpressing **(A)** NCI-ADR-RES and **(B)** KB-V-1 cancer cells were treated with DMSO (vehicle control) or branebrutinib at 1, 3, 5, or 10 μM for 72 h and processed for immunoblotting with indicated antibodies as described in Materials and methods. Representative Western blots (upper panel) and the corresponding quantification (lower panel) of P-gp are shown. α-Tubulin was used as an internal loading control. Values are presented as mean ± SD. Calculated from at least three independent experiments.

Next, studies have also reported that a drug-induced, transient down-regulation of a drug transporter is another mechanism to resensitize multidrug-resistant cancer cells to anticancer drugs (Cuestas et al., 2012; Natarajan et al., 2013). For that reason, we examined the protein expression of P-gp by immunoblotting with specific antibodies after treating P-gp-overexpressing NCI-ADR-RES and KB-V-1 cancer cells with branebrutinib (1–10 μM) for 72 h as described in Materials and methods. Our results show that the expression of P-gp at protein level in NCI-ADR-RES (Figure 3A) and KB-V-1 (Figure 3B) cancer cells was not significantly altered by branebrutinib over a period of 72 h, suggesting that branebrutinib reverses P-gp-mediated MDR in these cancer cell lines (Table 1) by blocking the drug efflux function of P-gp.

### Branebrutinib Enhances Colchicine-Induced Apoptosis in P-gp-Overexpressing Cancer Cells

**FIGURE 4:**
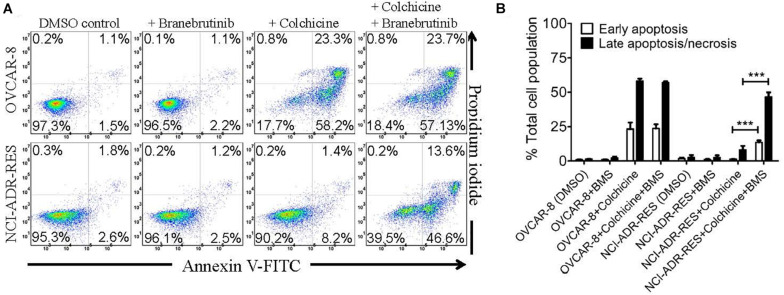
Branebrutinib enhances colchicine-induced apoptosis in P-glycoprotein (P-gp)-overexpressing cancer cells. Drug-sensitive OVCAR-8 and the P-gp-overexpressing multidrug-resistant NCI-ADR-RES cancer cells were treated with DMSO (control), branebrutinib at 20 μM (+BMS), colchicine at 500 nM (+ colchicine), or a combination of colchicine and branebrutinib (+colchicine + BMS) for 48 h, processed and analyzed by flow cytometry as described in Materials and methods. Representative flow cytometric dot plots are shown **(A)** and the corresponding quantification **(B)** are presented as mean ± SD. Calculated from at least three independent experiments. ***p < 0.001, versus the same treatment in the absence of branebrutinib.

To exclude the potential growth retardation effect of branebrutinib on P-gp-overexpressing multidrug-resistant cancer cells, we tested the effect of branebrutinib on drug-induced apoptosis in P-gp-overexpressing cancer cells. In addition to being a known drug substrate of P-gp (Kartner et al., 1983), colchicine is also a known inducer of apoptosis (Riordan and Ling, 1979). OVCAR-8 and NCI-ADR-RES cancer cells were treated with 0.5 μM of colchicine in the presence of DMSO (control) or 20 μM of branebrutinib for 48 h and proceed as described in Materials and methods. As expected, we found that colchicine induced the level of apoptosis in drug-sensitive parental OVCAR-8 cancer cells, from approximately 3% basal level to 82% of total apoptosis (Figure 4A, upper panels). In contrast, due to the activity of P-gp, the apoptosis-inducing effect of colchicine in P-gp-overexpressing NCI-ADR-RES cancer cells was significantly less (Figure 4A, lower panels). More importantly, we found that branebrutinib by itself does not induce substantial apoptosis in either cell line, however, it significantly enhanced colchicine-induced apoptosis in NCI-ADR-RES cancer cells, from 10 to 60% of early and late apoptosis (Figure 4B).

### Branebrutinib Stimulates ATPase Activity of P-gp

**FIGURE 5:**
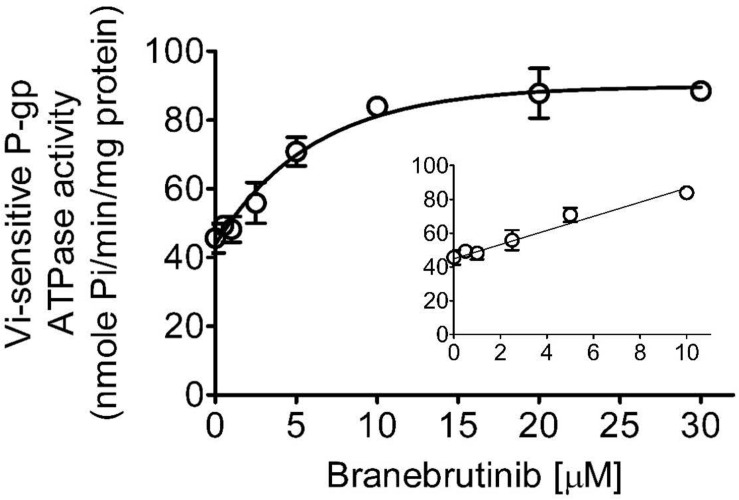
Branebrutinib stimulates the ATPase activity of P-glycoprotein (P-gp). The effect of 0–30 μM of branebrutinib (0–10 μM, inset) on P-gp-mediated ATP hydrolysis was measured in the membrane vesicles prepared from High-Five insect cells overexpressing human P-gp and recorded as vanadate (Vi)-sensitive ATPase activity as previously described (Wu et al., 2013). Points, mean from at least three independent experiments; bars, SD.

Knowing that the transport activity of P-gp is coupled to ATP hydrolysis (Ambudkar et al., 1999, 2003), the effect of branebrutinib on its ATP hydrolysis was examined to gain additional biochemical information on the interactions between branebrutinib and P-gp. As shown in Figure 5, branebrutinib stimulated Vi-sensitive ATPase activity of P-gp in a concentration-dependent manner, with maximum stimulation of almost 85% higher than the basal level of 45.6 ± 4.3 nmole P_*i*_/min/mg protein, and the half maximal effective concentration (EC_50_) value (concentration for branebrutinib to obtain 50% of maximum stimulation of P-gp ATPase activity) of approximately 4.5 μM. Furthermore, to determine whether branebrutinib and verapamil interact at the same substrate site of P-gp, we examined the effect of branebrutinib on verapamil-stimulated ATPase activity of P-gp. We found that branebrutinib had no significant effect on the ATPase activity stimulated by 5 μM of verapamil (Supplementary Figure 1). These results suggested that branebrutinib does not interact at the same P-gp substrate binding site as verapamil.

### Docking Analysis of Branebrutinib Binding to the Drug-Binding Pocket of P-gp

**FIGURE 6:**
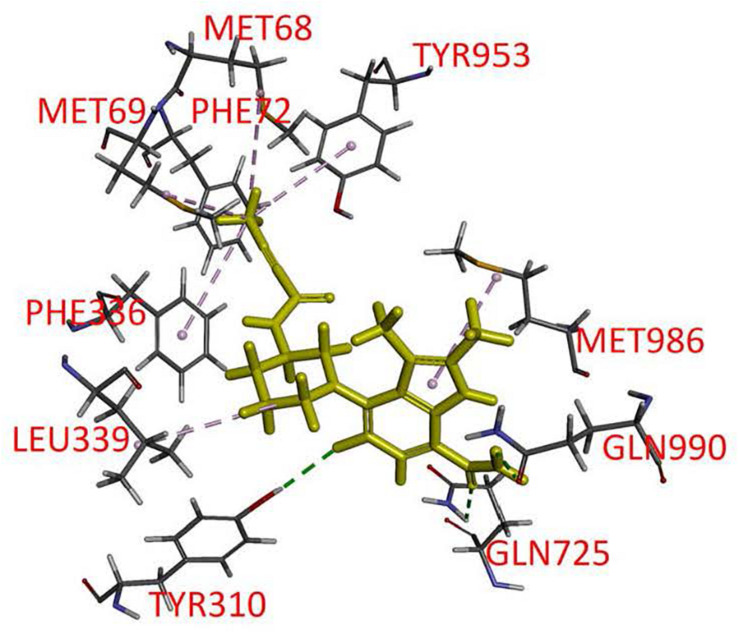
Docking of branebrutinib in the drug-binding pocket of P-glycoprotein (P-gp). Binding modes of branebrutinib with the protein structure of P-gp (PDB: 6QEX) was predicted by Accelrys Discovery Studio 4.0 software as described in Materials and methods. Branebrutinib is shown as a molecular model with highlighted yellow color and the atoms for interacting amino acid residues were colored as oxygen-red, nitrogen-blue, hydrogen-light gray and carbon- gray. Proposed interactions are presented as dotted lines.

Branebrutinib was docked into the human P-gp model based on cryo-EM structure (pdb.6QEX) and the binding was predicted to take place in the drug-binding cavity in the transmembrane region. The best binding conformation was selected with the binding energy calculated to be −52.83 kcal/mol. The residues from transmembrane helix (TMH) 1, 5, 6, 7, 11, and 12 were found to interact with branebrutinib. Most amino acids in the binding site interact with branebrutinib via hydrophobic interactions. The residues Met68, Met69, Phe72, Phe336, and Tyr953 of TMH 1, 6, and 11, respectively were predicted to interact with the propynyl, the residue Leu339 of TMH 6 with piperidine ring, and the indole moiety was predicted to interact with Met986 of TMH12 of P-gp. Hydrogen bonds were also found between Gln990 of TMH 12 and Gln725 of TMH7 with the amide moiety on 7-indole position of branebrutinib (Figure 6).

### P-gp-Overexpressing Cells Are Not Resistant to Branebrutinib

P-glycoprotein is known to mediate the transport of many TKIs (Eechoute et al., 2011; Tang et al., 2011, 2012; Robey et al., 2018; van Hoppe et al., 2019) and confer resistance to some of these TKIs (Mahon et al., 2003, 2008; Hiwase et al., 2008), including the BTK inhibitor ibrutinib (van Hoppe et al., 2018). To this end, we examined whether P-gp-overexpressing cells are less susceptible to branebrutinib treatment by determining the cytotoxicity of branebrutinib in multiple pairs of drug-sensitive parental cell lines and respective P-gp-overexpressing multidrug-resistant cell lines. As shown in Table 3, branebrutinib is equally cytotoxic to P-gp-overexpressing human ovarian NCI-ADR-RES cancer cells, human epidermal KB-V-1 cancer cells, human K562/i-S9 chronic myelogenous leukemia cells, and the corresponding drug-sensitive parental OVCAR-8, KB-3-1, and K562 cells. In addition, P-gp-transfected NIH3T3-G185 mouse fibroblast cells and MDR19-HEK293 cells, and the corresponding parental NIH3T3 and pcDNA3.1-HEK293 cells are also equally sensitive to branebrutinib treatment.

**TABLE 3:** Cytotoxicity of branebrutinib in drug-sensitive and P-glycoprotein (P-gp)-overexpressing multidrug-resistant cell lines.

Cell line	Type	Transporter expressed	IC_50_ (μM) ^†^	RF^‡^
OVCAR-8	Ovarian	–	39.28 ± 11.30	1.0
NCI-ADR-RES	Ovarian	P-gp	50.08 ± 17.26	1.3
KB-3-1	Epidermal	–	21.27 ± 8.45	1.0
KB-V-1	Epidermal	P-gp	26.35 ± 9.62	1.2
K562	Leukemia	–	57.66 ± 29.71	1.0
K562/i-S9	Leukemia	P-gp	54.11 ± 25.77	0.9
NIH3T3	–	–	23.59 ± 5.10	1.0
NIH3T3-G185	–	P-gp	29.41 ± 12.29	1.2
pcDNA3.1-HEK293	–	–	41.39 ± 16.61	1.0
MDR19-HEK293	–	P-gp	47.17 ± 16.21	1.1

*RF, resistance factor.*

*^†^IC_50_ values were calculated from at least three independent experiments as described in Materials and methods.*

*^‡^RF value was obtained by dividing the IC_50_ value of branebrutinib in P-gp-overexpressing cells by the IC_50_ value of branebrutinib in respective parental cells.*

## Discussion

Cancer patients with poor response to conventional cytotoxic anticancer drugs, associated with the overexpression of P-gp in cancer cells, remains a major challenge in cancer chemotherapy (Gillet and Gottesman, 2010; Robey et al., 2018). While there is no effective and clinically safe inhibitors of P-gp available to date (Robey et al., 2018; Leopoldo et al., 2019; Dong et al., 2020), the results of several clinical trials have shown that cancer patients could benefit from the co-administration of conventional anticancer agents with TKIs (Geyer et al., 2006; Moore et al., 2007; Yang et al., 2013; Cetin et al., 2014; Alemany et al., 2018). Results of the combination therapy trial of gemcitabine and erlotinib were much better than monotherapy with gemcitabine in advanced pancreatic cancer patients (Moore et al., 2007; Yang et al., 2013). Similarly, results of combination therapy trial of capecitabine with lapatinib were significantly better than monotherapy with capecitabine in patients with human epidermal growth factor receptor 2 (HER2)-positive advanced breast cancer (Geyer et al., 2006; Cetin et al., 2014). More recently, encouraging results were reported in a combination therapy trial of doxorubicin with nilotinib, used to inhibit the activity of P-gp, in patients with sarcomas (Alemany et al., 2018). Together, these studies indicate that further investigation into combination therapies of multidrug-resistant cancers using conventional anticancer drugs and TKIs as modulators of P-gp is warranted. Consequently, we and others have been exploring the possibility of exploiting the polypharmacology properties of these TKIs for an additional mode of action against P-gp (Hsiao et al., 2016, 2019; Zhang et al., 2017; Wu and Fu, 2018; Wu et al., 2019, 2020a,2020b).

**FIGURE 7:**
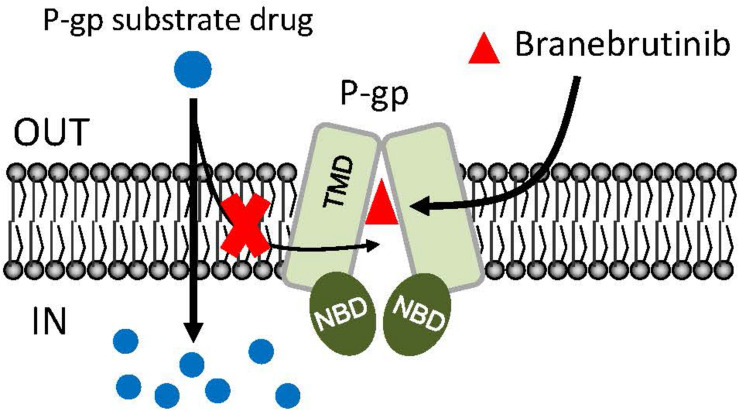
Simplified schematic of branebrutinib resensitizing P-glycoprotein (P-gp)-overexpressing multidrug-resistant cancer cells to anticancer drugs by blocking the drug efflux function of multidrug transporter. The intracellular concentration of a P-gp substrate drugs (blue circles) in P-gp-overexpressing cells is significantly reduced by the drug efflux function of P-gp. However, in the presence of branebrutinib (red triangles), branebrutinib outcompetes the binding of P-gp substrate drug to the same drug-binding pocket of P-gp and consequently restores the intracellular accumulation and efficacy of substrate drugs in P-gp-overexpressing multidrug-resistant cells.

In the current study, we investigated the *in vitro* chemosensitizing effect of a BTK inhibitor branebrutinib in P-gp-overexpressing multidrug-resistant cancer cells. We determined the intrinsic toxicity of branebrutinib in several pairs of drug-sensitive and P-gp-overexpressing multidrug-resistant cell lines. Although P-gp is known to mediate resistance to numerous TKIs (Mahon et al., 2003; Hiwase et al., 2008), we found that P-gp does not confer resistance to branebrutinib in these cell lines (Table 3). Our results suggest that branebrutinib is not rapidly pumped out of cancer cells by P-gp and that the overexpression of P-gp is not likely to contribute significantly to the development of branebrutinib resistance in patients. Nevertheless, the mechanisms of resistance to branebrutinib in patients remain to be determined in clinical studies. Next, we examined the effect of branebrutinib, at sub-toxic concentrations, on P-gp-mediated resistance to anticancer drugs vincristine, paclitaxel, and colchicine. We found that branebrutinib resensitizes P-gp-overexpressing multidrug-resistant KB-V-1 and NCI-ADR-RES cancer cells, as well as HEK293 cells transfected with human P-gp, to these anticancer drugs in a concentration-dependent manner (Figure 1, Tables 1, 2). The results of branebrutinib inhibiting P-gp-mediated drug efflux (Figure 2) without altering the protein expression of P-gp (Figure 3) in KB-V-1 and NCI-ADR-RES cancer cells suggest that branebrutinib reverses P-gp-mediated MDR by blocking the drug transport function of P-gp and consequently restores the susceptibility of P-gp-overexpressing multidrug-resistant cancer cells to drug-induced apoptosis (Figure 4). These results, together with the P-gp-specific ATPase data (Figure 5) and the *in silico* docking analysis of branebrutinib binding to the substrate-binding site of P-gp in the inward-open conformation (Figure 6), indicate that this compound attenuates the binding of another drug substrate by interacting with numerous amino acid residues within the drug-binding pocket of P-gp (Figure 7).

In summary, we demonstrated that branebrutinib could effectively reverse P-gp-mediated MDR in cancer cells by modulating the activity of P-gp. Although the possibility of other mechanisms contributing to the resensitization of multidrug-resistant cancer cells remains, and that unexpected adverse drug interactions may occur in combination therapies (Stewart et al., 2004; Shukla et al., 2008; Libby and Hromas, 2010; Robey et al., 2018), we report here an additional action of branebrutinib that could be utilized in combination therapy with conventional anticancer drugs to treat multidrug-resistant cancers associated with the overexpression of P-gp, which should be investigated further.

## Data Availability Statement

The raw data supporting the conclusions of this article will be made available by the authors, without undue reservation.

## Author Contributions

C-PW, T-HH, Y-SW, and SA designed the experiments. C-PW, Y-SW, and SA wrote the original draft manuscript and analyzed the data. C-PW and SA reviewed the manuscript. C-PW, MM, Y-SW, Y-CC, S-HH, and Y-HH performed the experiments. C-PW and SA edited the manuscript. All authors discussed the data and approved the final manuscript.

## Conflict of Interest

The authors declare that the research was conducted in the absence of any commercial or financial relationships that could be construed as a potential conflict of interest.
