# Supplementary material for: Branebrutinib (BMS-986195), a Bruton’s Tyrosine Kinase Inhibitor, Resensitizes P-Glycoprotein-Overexpressing Multidrug-Resistant Cancer Cells to Chemotherapeutic Agents
Source: Front Cell Dev Biol. 2021 Jul 19;9:699571. doi: 10.3389/fcell.2021.699571 (PMC8326665; doi:10.3389/fcell.2021.699571)
Supplement: Supplementary file 1 [file Data_Sheet_1.PDF]

Supplementary Material

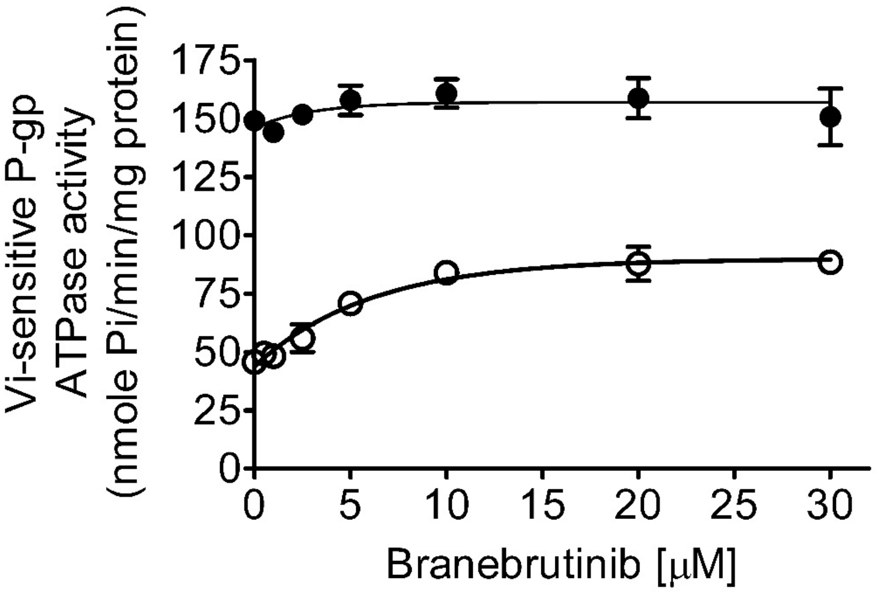

Supplementary Figure 1. The effect of branebrutinib (0 – 30 μM) on the basal ATPase activity of P-gp (open circles) and verapamil (5 μM)-stimulated ATPase activity of P-gp (filled circles) was measured in the membrane vesicles prepared from High-Five insect cells over-expressing human P-gp and recorded as vanadate (Vi)-sensitive ATPase activity. Points, mean from at least three independent experiments; bars, S.E.M.
